# Pulmonary manifestations of systemic lupus erythematosus patients with and without antiphospholipid syndrome

**DOI:** 10.12669/pjms.311.6544

**Published:** 2015

**Authors:** Muhammad Afzal Hamdani, Abdul Rahman Saud Al-Arfaj, Khalid Parvez, Faisal Naseeb, Abdalla El Fateh Ibrahim, Joseph Hope Cal

**Affiliations:** 1 Muhammad Afzal Hamdani, FCPS, Dip-Card., MRCP. Senior Registrar, Division of Rheumatology, Department of Medicine, King Khalid University Hospital (KKUH), King Saud University (KSU), Riyadh, Saudi Arabia.; 2 Abdul Rahman Saud Al-Arfaj, MRCP(UK), FRCPC, Professor of Rheumatology, American Board of Internal Medicine, American Board of Rheumatology. Department of Medicine, King Khalid University Hospital (KKUH), King Saud University (KSU), Riyadh, Saudi Arabia.; 3 Khalid Parvez, FCPS. Registrar, Division of Rheumatology, Department of Medicine, King Khalid University Hospital (KKUH), King Saud University (KSU), Riyadh, Saudi Arabia.; 4 Faisal Naseeb, FCPS. Registrar, Division of Rheumatology, Department of Medicine, King Khalid University Hospital (KKUH), King Saud University (KSU), Riyadh, Saudi Arabia.; 5 Dr. Abdalla El Fateh Ibrahim, MRCP. Assistant Professor of Pulmonology, Department of Medicine, King Khalid University Hospital (KKUH), King Saud University (KSU), Riyadh, Saudi Arabia.; 6 Joseph Hope Cal, FPCP, FPCCP. Registrar, Division of Pulmonology, Department of Medicine, King Khalid University Hospital (KKUH), King Saud University (KSU), Riyadh, Saudi Arabia.

**Keywords:** Systemic Lupus Erythematosus, Antiphospholipid Syndrome, Pulmonary manifestations. High resolution computed tomography

## Abstract

**Objective:**

To uncover the pulmonary manifestations of Systemic Lupus Erythematosus (SLE) patients alone and to compare findings with antiphospholipid syndrome (APS) associated with SLE.

**Methods:**

This cross sectional comparative study was carried out at King Khalid University Hospital (KKUH)/King Saud University (KSU), a tertiary care hospital, Riyadh, Kingdom of Saudi Arabia. From June 2012 to March 2014, 96 diagnosed SLE patients with respiratory symptoms were included in the study and divided into two groups. Group one included SLE without antiphospholipid syndrome (APS) and group two SLE with APS. We compared Demographic features, clinical manifestations and findings of chest X-Ray, Arterial Blood Gases, Pulmonary function tests, six minute walk test, ventilation perfusion scan, echocardiography and chest high resolution computed tomography.

**Results:**

Demographic and clinical characteristics of two groups were similar. Previous history of deep venous thrombosis (3% vs 27.6%, p=0.001), pulmonary embolism (3% vs34.5%, p<0.0001) and abortions (7.5% vs 27.6%, p=0.019) were significantly more in group two. Levels of Anticardiolipin antibody (0% vs 100%, p<0.0001) and lupus anticoagulant (1.5% vs 79.3%, p<0.0001) were also significantly higher in group two. Hypoxemia measured by pulse oximetry (43.3% vs 65.5% p=0.045, pulmonary Arterial Hypertension (15.5% vs 39.3% p=0.014)), and pulmonary embolism (3.4% vs 21.4% p=0.013) and ventilation perfusion mismatch on V/Q scan (1.5% vs 24.1% p=0.001) were more frequent in group two.

**Conclusion:**

Hypoxemia, pulmonary embolism and pulmonary arterial hypertension were significantly high in SLE patients with APS, requiring long term anticoagulation and treatment and close follow-up.

## INTRODUCTION

Systemic lupus erythematosus (SLE) is an autoimmune disease characterized by multiorgan involvement like kidneys, joints, lungs, central nervous system as well as hematopoietic system. It is characterized by disturbances and interplay of various factors in immune system, heritable factors, hormonal features and environmental factors. Features also vary with characteristics such as age, race, gender, ethnicity, and country of birth.^1^ The disease is most common among women of child bearing age. In Saudi Arabia prevalence of SLE is estimated to be 19.28 per 100,000 population.^2^

Lungs are commonly involved among the other organs. Damage and dysfunction is mediated by autoantibodies and immune complex formation. At some time during the disease course, about 50% of patients with systemic lupus erythematosus show signs of involvement of the lung, its vasculature, the pleura, and diaphragm.^3^ Pleuritic chest pain, coughing, and shortness of breath are often the first clues to the lung involvement of SLE itself.^4^

Prevalence of antiphospholipid syndrome (APS), in SLE is variable in different areas. Presence of antibodies, one of the criteria defining APS was IgG 49.7%, IgM 33.5% and lupus anticoagulant 27.0% from Saudi Arabia.^5^ Prevalence of APS secondary to SLE is 25.38% in India.^6^ The APS is a systemic autoimmune disorder associated with hypercoagulabilty, pregnancy morbidity, thrombocytopenia, and thromboembolic phenomenon. Pathogenic antiphospholipid antibodies cause vascular thrombosis, recurrent miscarriages, thrombocytopenia and livido reticularis.^7^ A broad spectrum of pulmonary disorders may occur in primary or secondary antiphospholipid syndrome, the common being pulmonary thromboembolism, pulmonary hypertension, intra-alveolar haemorrhage and fibrosing alveolitis.^8^^,^^9^

To the best of our knowelege there has been no previous study in our region that sought to explore differences between pulmonary manifestations of SLE and SLE with APS patients, presenting with respiratory symptoms. Knowing that APS is a hypercoagulable state we expect complication like pulmonary embolism and pulmonary hypertension with its sequelae to be seen more in SLE with APS patients. In this study we sought to determine the usual pulmonary manifestations in two groups. In addition, we wanted to see important differences among two groups.^10^

## METHODS

This was a cross sectional comparative study conducted in King Khaled University Hospital/King Saud University from June 2012 to March 2014. A total of ninety six (96) patients, seen in the rheumatology out-patient clinic or admitted in the hospital with a diagnosis of SLE or SLE with APS were included. Diagnosis of SLE was made according to the American College of Rheumatology (ACR) criteria for classification of SLE,.^11^ while diagnosis of APS was made according to the international consensus statement on an update of classification criteria for APS.^12^ Patients presented with respiratory symptoms were included in the study. These patients were divided into two groups, group one SLE (n=67) and group two SLE with APS (n=29) patients. We excluded patients with any other connective tissue disease and pregnant females.

Sample size was calculated, using G*Power Version 3.1.9.2 the total sample size was computed at 108, with error probability set at 0.05, and power set at 0.8. Since we were only able to come up with 96 subjects, the actual power of the study is 0.75.^13^

A detailed assessment form was utilized to collect the demographic and clinical data of the patients. Complete history and physical examination was performed and relevant investigations were ordered. Involvement of other organs such as kidneys, brain, skin, joints, hematological and gastrointestinal was also noted. Complete blood count, erythrocyte sedimentation rate, C- reactive proteins, coagulation profile, renal function tests, liver function tests, urine analysis and autoantibody profiles were taken in all patients. Where indicated after doing renal biosy results were also obtained. Arterial blood gases (ABGs), pulmonary function tests (PFTs), and six minute walk distance (6MWDT) was taken to establish functional capacity. Plain chest x-rays, high resolution computed tomography (HRCT) and where indicated ventilation perfusion scan (V/Q scan) and echocardiography were taken. Pulmonary hypertension was measured and interpreted based on the diameter of the main pulmonary arteries and its peripheral branches on CT angiography^14^ and on echocariography by applying the method of differential pressure of tricuspid regurgitation, pulmonary artery dilatation and right ventricular enlargement.^15^

***Statistical analysis: ***Descriptive statistics (means, standard deviation, and percentages) were used to describe the quantitative and categorical study variables. Chi- square statistics and the Fisher’s exact test were used for categorical data. A two-sided p <0.05 was considered statistically significant. SPSS version 18 (SPSS inc. Chicago, IL, USA) was used for all analysis.

## RESULTS

The demographic features and clinical profile of group one: SLE (n=67), group two: SLE with APS (n=29) are shown in Table-I. There was no significant differences in the demographic characteristics of both groups.

The most common presenting complaint of both groups were cough, dyspnea, chest pain, fever, hemoptysis and palpitation. The occurrence of fever was found to be significantly higher in group one (34.3% vs 10.3%, p = 0.015) compared to group two. Physical examination revealed hypoxemia on pulse oximetry, chest crackles and rhonchi, bronchial breathing, chest dullness, pleural rub and edema were common findings in both groups with the finding of hypoxemia significantly more common in group two (43.3% vs 65.5%, p = 0.011). Common findings on other systems examination included oral ulcers, arthritis, skin rash, lymph node enlargement, serositis with oral ulcers significantly more common in group two (37.3% vs 65.5% p = 0.011) Table-I. 

Review of the past medical history of the patients revealed no significant differences except for in group two the high prevalence of the deep venous thrombosis (3% vs 27.6%, p = 0.001), as well as pulmonary embolism (3% vs 34.5%, p< 0.0001) and spontaneous abortion (7.5% vs 27.6%, p = 0.019) as compared to group one Table-II.

The autoanibody profile of both groups were similar except for the significantly elevated anticardiolipin antibodies (0% vs 100%, p < 0.0001) and lupus anticoagulant (1.5% vs 79.3%, p < 0.0001) in group two as compared to group one. Among both groups patients who underwent kidney biopsy, lupus nephritis Class II (30.3% vs 58.8%, p = 0.032) and class III was (18.2% vs 52.9%, p = 0.011) were more common in group two, while class IV lupus nephritis (60.6% vs 17.6%, p = 0.004) was more common in group one (Table-III). 

The results of pulmonary investigations are shown in Table-IV. Hypoxemia on arterial blood gas (ABG) analysis showed no significant differences between two groups. On chest x-ray, most common findings were lower zone infiltrates, consolidation, pleural effusion and/or thickening. For the pulmonary function tests, diminished forced vital capacity (FVC), total lung capacity (TLC), and diffusion capacity for carbon monoxide (DLco) were seen in both groups. For those who underwent HRCT, the most common findings were ground glass opacities, honeycombing, pleural effusion/thickening, atelectasis and hilar lymphadenopathy. Pulmonary embolism (3.4% vs 21.4%, p = 0.013) and changes ascribed to pulmonary hypertension (15.5 vs 39.3%, p = 0.014) were more commonly found in group two. Those patients who underwent V/Q scan, mismatch was more in patients of group two than of group one (1.5% vs 24.1%, p = 0.001). Pulmonary hypertension measured by transthoracic echocardiography, was also significantly higher in group two (34.5% vs 80.8%, p < 0.0001).

## DISCUSSION

To the best of our knowledge this is the first study in this region comparing pulmonary manifestations of patients with respiratory symptoms having SLE with or without APS. Most of the other studies in literature addressed lung involvement only in early SLE patients or SLE with APS who were asymptomatic.^16^^,^^17^

In this study respiratory symptoms like cough, shortness of breath, chest pain and others were almost comparable in both group of patients. Previous history of deep venous thrombosis (3% vs 27.6%, p = 0.001), pulmonary embolism (3% vs 34.5%, p < 0.0001) and abortions (7.5% vs 27.6%, p = 0.019 were significantly more in group two. This is compatible with studies done by Bigioggero H^18^, in which it has been shown that association between SLE and APS patients have increased risk of thromboembolism venous, arterial and pulmonary as compared to SLE patients alone. Physical findings like crepitations, chest dullness, rhonchi, bronchial breathing, and pleural rub were not significantly different, except hypoxemia on pulse oximetry (43.3% vs 65.5%) in two groups, (p *= *0.045) and on arterial blood gas analysis hypoxemia was (83.6% vs 96.6%, p = 0.099). The cause of hypoxemia in patients in group one patients might be because all patients had respiratory symptoms and pathology like pleural effusion/thickening, consolidation leading to shunting of blood, pneumonitis, atelectasis, pulmonary hemorrhage, infections and low lung volume were common findings. Another cause may be acute reversible hypoxemia syndrome and pulmona ry venoocclusive disease. These in group one patients was similar, but percentage of these findings was higher than a study done by Paran D.^19^ A recent study done in Greece by Tzouvelekis,^20^ showed HRCT findings such as nonspecific interstitial pneumonia (NSIP) and usual interstitial pneumonitis (UIP) pattern was about 33% in patients with APS patients. Other findings like acute pneumonitis, ground glass opacities, basal atelectasis, pleural effusion, pleural thickening, pulmonary artery thrombosis and embolism were present in less number of patients in this study as compared to ours. In our study findings of pulmonary hypertension, and pulmonary thromboembolism were higher in group two, (p = 0.014 and 0.013 respectively). Twenty percent of these patients had honey combing, bronchiactasis and cystic changes and only 17% had findings consistant with shrinking lung syndrome.

Pulmonary function test results in our patients showed decreased FVC followed by TLC and decreased DLco in majority of the patients, however decrease was much higher than review done in a study by Mittoo S.^21^ As shown in Table-IV those patients who underwent V/Q scan in both groups it was normal in most of the patients, however in group two 24.1% of the patients were showing the mismatch in ventilation and perfusion showing the high probability of pulmonary embolism in this group of patients.

**Table-I:** Demographic and clinical profile of patients in two groups

**Variable**	**Group One SLE(=67)**	**Group Two** **SLE +APS (N=29)**	**p-value**
Age, years	38.4±14.0	37.1±10.2	0.654
Gender			0.446
Female, n (%)	62 (92.5)	25 (86.2)	
Male, n (%)	5 (7.5)	4 (13.8)	
Smoking, n (%)	7 (10.4)	4 (13.8)	0.730
Disease duration, months	88.5±70.4	92.3±74.1	0.813
Presenting symptoms.			
Cough	48 (71.6)	15 (51.7)	0.059
Dyspnea	59 (88.1)	25 (86.2)	0.750
Chest pain, pleuritic	18 (26.9)	13 (44.8)	0.100
Chest pain, non-pleuritic	15 (22.4)	4 (13.8)	0.412
Fever	23 (34.3)	3 (10.3)	0.015*
Hemoptysis	11 (16.4)	2 (6.9)	0.332
Palpitations	18 (26.9)	11 (37.9)	0.278
Physical Examination			
Hypoxemiaǂ	29 (43.3)	19 (65.5)	0.045*
Raised JVP	6 (9.0)	5 (17.2)	0.299
Clubbing	4 (6.0)	1 (3.4)	1.000
Edema	14 (20.9)	7 (24.1)	0.724
Pleural rub	23 (34.3)	12 (41.4)	0.510
Crackles	56 (83.6)	24 (82.8)	1.000
Chest dullness	29 (43.3)	18 (62.1)	0.091
Bronchial breathing	14 (20.9)	4 (13.8)	0.413
Rhonchi	27 (40.3)	10 (34.5)	0.591
Raynaud’s phenomenon	3 (4.5)	2 (6.9)	0.636
Other systemic examination			
Arthritis	57 (85.1)	21 (72.4)	0.144
Oral ulcer	25 (37.3)	19 (65.5)	0.011*
Skin rash	49 (73.1)	19 (65.5)	0.451
Focal neurologic deficit	4 (6.0)	0	0.312
Lymph node	18 (26.9)	6 (20.7)	0.521
Livido reticularis	6 (9.0)	4 (13.8)	0.484
Serositis	25 (37.3)	10 (34.5)	0.791

Data presented as mean ± SD and n(%)

* P- Value < 0.05

ǂ Based on pulse oximetry

**Table-II:** Review of past history between two groups

**Variable**	**Group One** **SLE (n=67)**	**Group Two** **SLE + APS (n=29)**	**p-value**
Past History			
Heart Failure	4 (6.0)	1 (3.4)	1.000
Bronchial Asthma	4 (6.0)	1 (3.4)	1.000
Chronic Obstructive Pul. Disease	12 (17.9)	3 (10.3)	0.542
Deep Vevous Thrombosis	2 (3.0)	8 (27.6)	0.001*
Pulmonary embolism	2 (3.0)	10 (34.5)	< 0.0001*
Vasculitis	16 (23.9)	7 (24.1)	0.978
Central Nervous System	12 (17.9)	2 (6.9)	0.216
Lupus Nephritis	25 (37.3)	12 (41.4)	0.707
Gastrointestinal System	11 (16.4)	4 (13.8)	1.000
Skin involvement	28 (41.8)	14 (48.3)	0.556
Blood Abnormalities	26 (38.8)	12 (41.4)	0.813
History of Abortion	5 (7.5)	8 (27.6)	0.019*
Serositis	15 (22.4)	9 (31.0)	0.369

Data presented as n (%)

* P -value < 0.05

**Table-III:** Autoantibody profiles and type of lupus nephritis in two groups

**Variable**	**Group one** **SLE (n=67)**	**Group Two** **SLE + APS (n=29)**	**p-value**
ANA	66 (98.5)	29 (100)	1.000
Anti-dsDNA	53 (79.1)	26 (89.6)	0.214
Anti-Sm	22 (32.8)	15 (51.7)	0.081
Anti-Ro	23 (34.3)	12 (41.4)	0.510
Anti-La	19 (28.4)	8 (27.6)	0.938
ACL Ab	0	29 (100)	0.0001*
LAC	1 (1.5)	23 (79.3)	0.0001*
Anti-RNP	6 (9.0)	6 (20.7)	0.175
Anti-histone	0	1 (3.4)	0.295
Rheumatoid factor			0.267
Not done	0	5 (17.2)	
Negative	44 (65.7)	16 (55.1)	
Positive	21 (31.3)	8 (27.5)	
Lupus nephritis	(N=33)	(N=17)	
Class I	1 (3)	1 (5.9)	1.000
Class II	10 (30.3)	10 (58.8)	0.032
Class III	6 (18.2)	9 (52.9)	0.011
Class IV	20 (60.6)	3 (17.6)	0.004
Class V	2 (6.1)	1 (5.9)	1.000

Data presented as n (%)

* P -Value < 0.05.

**Table-IV:** Pulmonary manifestations of SLE and SLE with APS patients

**Variable**	**Group One** **SLE (n=67)**	**Group Two** **SLE + APS (n=29)**	**p-value**
Hypoxemia ǂ	56 (83.6)	28 (96.6)	0.099
Type 1 respiratory failure	33 (49.2)	15 (51.7)	0.824
Chest x-ray			
Normal	8 (11.9)	4 (13.8)	0.750
Lower zone infiltrates	37 (55.2)	17 (58.6)	0.758
Pleural effusion	28 (41.8)	16 (55.2)	0.227
Consolidation	6 (9.0)	4 (13.8)	0.484
Pleural thickening	23 (34.3)	6 (20.7)	0.181
Alveolar shadows	5 (7.5)	2 (6.9)	1.000
Low lung volume	19 (28.4)	5 (17.2)	0.248
Cardiomegaly	11 (16.4)	5 (17.2)	1.000
Pulmonary function test	N=50	N=19	
Decreased FVC	48 (96)	19 (100)	1.000
Decreased TLC	38 (76)	14 (73.7)	1.000
Decreased DLCO	28 (56)	8 (42.1)	0.468
HRCT findings	N=58	N=28	
Ground glass opacities(UIP and NSIP)	22 (37.9)	9 (32.1)	0.600
Honeycombing, cystic changes and air trapping	20 (34.5)	5 (17.8)	0.112
Acute pneumonitis	9 (15.5)	4 (14.3)	1.000
Organizing pneumonia	8 (13.8)	4 (14.3)	1.000
Pleural effusion	25 (43.1)	15 (53.6)	0.362
Pleural thickening Infection	24 (41.4) 25(43.1)	7 (25) 8(28.5)	0.138 0.139
Atelectasis	22 (37.9)	8 (28.6)	0.393
“Shrinking lung syndrome”	10 (17.2)	6 (21.4)	0.640
Pulmonary hemorrhage	5 (8.6)	2 (7.1)	1.000
Pulmonary hypertension	9 (15.5)	11 (39.3)	0.014*
Pulmonary embolism	2 (3.4)	6 (21.4)	0.013*
Hilar lymphadenopathy	19 (32.8)	8 (28.6)	0.695
Ventilation-perfusion lung scan			0.001*
Not done	33 (49.2)	10 (34.5)	
Normal	33 (49.2)	12 (41.4)	
Abnormal	1 (1.5)	7 (24.1)	
Six-minute walk test			0.458
Not done	19 (28.4)	11 (37.9)	
Normal	11 (16.4)	6 (20.7)	
Abnormal	37 (55.2)	12 (41.4)	
Echocardiography	19 (34.5)	21 (80.8)	<0.0001*

Data presented as n (%)

* P- Value < 0.05

ǂ Based on arterial blood gas

Association between clinical features of APS and antiphospholipid antibodies, previous or recent history of deep venous thrombosis is strongly associated with pulmonary embolism. Pulmonary hypertension in our study is slightly higher as compared to other studies.^22^^,^^23^ Cystic lung changes were found only in one patient with SLE in our study as mentioned as case reports by Maeda.^24^ Shrinking lung syndrome (low lung volume), should be considered in patients who present with dysnoea. In literature shrinking lung syndrome in SLE may be under diagnosed, it should be suspected in SLE patients who present with dyspnea and x-ray findings of low lung volume, elevated diaphragm and restrictive pattern of PFTs, without any other apparent cause.^25^

In conclusion there was no marked difference in the pulmonary findings in both groups apart from hypoxemia, pulmonary thromboembolism and pulmonary hypertension, which was more in group two patients. So long term anticoagulation is needed in SLE with APS patients having pulmonary embolism to prevent mortality and its recurrence. Standard treatment is recommended according to appropriate clinical settings. However it is very important to rule out infections and even empirical antibiotic therapy should be given where infection is suspected.

## Author’s contribution:

**MAH:** Conception and design, data collection, analysis and interpretation of data, drafting the research article.

**ASAA:** Supervision, conception and design, analysis and interpretation of data, critically revising drafted work.

**KP and FN:** Facilitate data collection, analysis, critically revising draft work.

**AEI:** Review of all radiological procedures and analysis of pulmonary function tests and critically revising drafted work.

**JHC:** Facilitate statistical analysis of data, critically revising drafted work.

## Notes

***Source of funding:*** Nil.
